# Severe Lymphoma-Associated Hemophagocytic Syndrome in a Young Woman

**DOI:** 10.7759/cureus.53649

**Published:** 2024-02-05

**Authors:** Kabeer Ali, Justin Medina, Pasquale W Benedetto

**Affiliations:** 1 Internal Medicine, Eric Williams Medical Sciences Complex, Champs Fleurs, TTO; 2 Internal Medicine, Jackson Memorial Hospital, Miami, USA; 3 Hematology/Oncology, Jackson Memorial Hospital, Miami, USA

**Keywords:** hlh-94, idiopathic hlh, secondary hlh (shlh), primary hlh, reactive hlh, secondary hemophagocytic lymphohistiocytosis (hlh), hemophagocytic lymphohistiocytosis (hlh)

## Abstract

Hemophagocytic lymphohistiocytosis (HLH) is a rare, life-threatening hyperinflammatory syndrome characterized by profound immune system activation. In adults, most cases of HLH are due to an underlying pathology- such as infection, malignancy, or autoimmune disease. It is a disease that can progress to rapid clinical deterioration and be difficult to diagnose. Nevertheless, regardless of etiology, most patients with HLH benefit from treatment. This paper highlights the challenges involved in diagnosing and managing this condition in practice, with an emphasis on how young, previously healthy young adults can present in a critically ill state.

## Introduction

Hemophagocytic lymphohistiocytosis (HLH) is a rare hyperinflammatory syndrome of aggressive and excessive immune activation leading to end organ damage. It is more often seen in pediatric populations. However, based on retrospective analyses, HLH has been demonstrated in adults of all ages, in various ethnic groups and geographic locations [1]. Based on the North American Consortium for Histiocytosis (NACHO), HLH is most recently defined using the terminology of HLH syndrome (genetic defects of lymphocyte cytotoxicity), HLH disease (distinctive immune activation associated with specific genetic or environmental cause), and HLH disease mimics [2]. It is associated with a very high mortality rate, namely due to cytokine storms from the persistent activation of macrophages, natural killer cells, and cytotoxic T-lymphocytes.

Furthermore, the patient can be subacute, which may lead to a delay in diagnosis until the disease process has reached a catastrophic point. In this case, we describe a fairly subacute case with a month-long history of symptoms before presentation to the hospital. At this point, the patient was very ill due to excessive cytokine production. This case highlights the clinical features and rapid progression of this high-mortality condition, hoping that it can lead to quicker recognition and better patient outcomes.

## Case presentation

A 25-year-old Latin American woman with no past medical history was admitted to the hospital, endorsing a one-month history of worsening abdominal distension, jaundice, and intermittent non-cyclical chills. On arrival, her vital signs were within normal limits. However, her physical examination revealed an ill-appearing female with a grossly distended, tender abdomen, generalized jaundice, and bilateral inguinal lymphadenopathy.

Initial laboratory examinations revealed decreased white blood cell count (WBC) and hemoglobin (Table 1). A basic metabolic panel was within normal limits, but liver function tests were significantly deranged, with elevated transaminases and direct hyperbilirubinemia. Abnormal coagulation studies revealed elevated activated partial thromboplastin time (APTT) and international normalized ratio (INR). Significant elevations were also noted in lactate dehydrogenase (LDH) and ferritin. Epstein-Barr virus (EBV) IgG was positive, but IgM was negative. Of note, SARS-CoV-2, human immunodeficiency virus (HIV) rapid antigen, rapid plasma reagin (RPR), human T-lymphotropic virus (HTLV) 1 and 2, and hepatitis B and C surface antibodies were all negative.

**Table 1:** Laboratory values at presentation WBC: White blood cell count; AST: Aspartate transaminase; ALT: Alanine transaminase; APTT: Activated partial thromboplastin time; INR: International normalized ratio; LDH: Lactate dehydrogenase

Lab	Value	Reference Range
WBC	3.1 × 10^9^/L	4–11 x 10^9^/L
Hemoglobin	9.1 g/dL	11.6-15 g/dL
AST	332 U/L	8-33 U/L
ALT	329 U/L	7-56 U/L
Direct Bilirubin	2.51 mg/dL	<0.3 mg/dL
APTT	46 seconds	30-40 seconds
INR	1.4	<1.1
LDH	2228 units/L	105-233 units/L
Ferritin	130000 ng/ml	12-150 ng/ml
Triglycerides	632 mg/dL	<150 mg/dL

**Figure 1:**
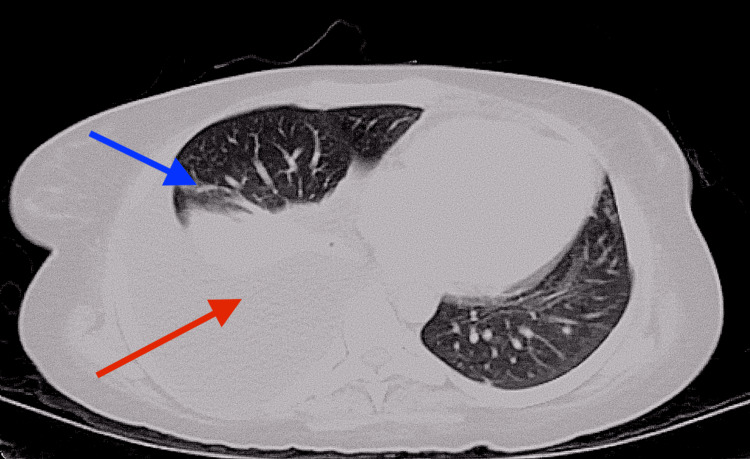
Multiple ground glass opacities (blue arrow), with large right pleural effusion (red arrow)

**Figure 2:**
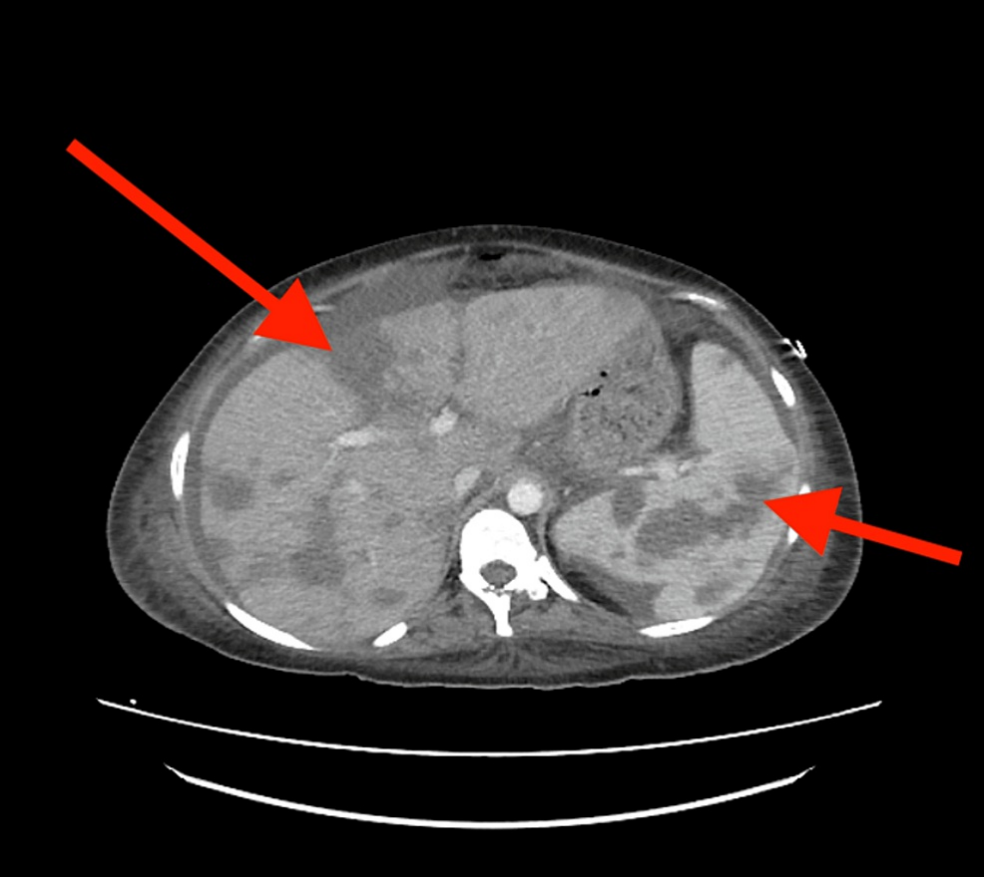
Numerous hypoattenuating lesions throughout the liver and spleen (red arrows)

**Figure 3:**
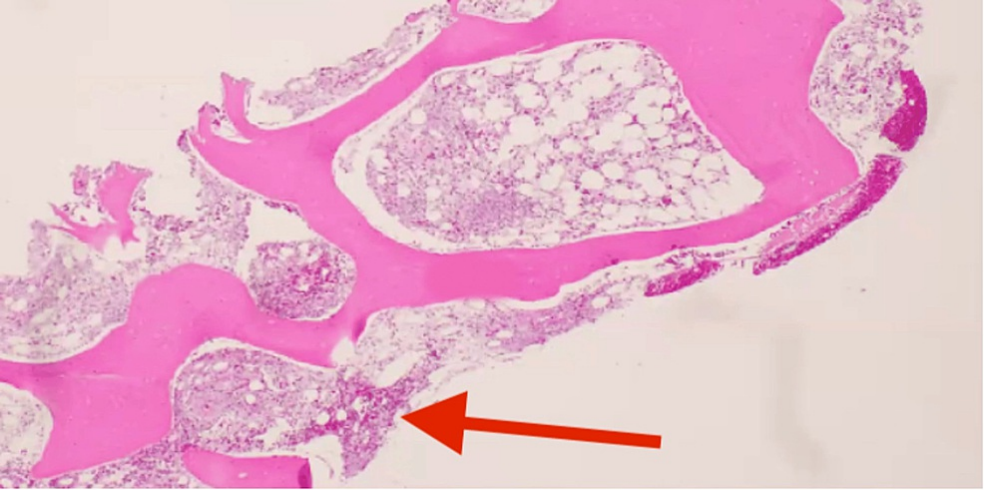
Hypocellular bone marrow, with the red arrow highlighting significant hemophagocytosis and atypical lymphoid proliferation

A contrast-enhanced computed tomography scan of the chest, abdomen, and pelvis was performed for further evaluation, demonstrating multiple ground-glass/nodular opacities in both lungs and bilateral pleural effusions in the right more significant than the left (figure 1). Numerous hypoattenuating lesions were seen throughout the liver and spleen, with retroperitoneal, right external iliac, and right inguinal lymphadenopathy (figure 2). Multiple biopsies were performed to elucidate a pathological diagnosis. An inguinal node biopsy showed atypical vascular lymphohistiocytic proliferation, with positivity for CD68, CD20, and CD5, suggesting a B-cell lymphoma. A liver biopsy showed extensive hepatic necrosis, but rare hemophagocytosis was present. Finally, a bone marrow biopsy showed a hypocellular bone marrow (ten percent) with significant hemophagocytosis and atypical lymphoid proliferation (figure 3). Flow cytometry of the bone marrow revealed no increase in blasts and no immunophenotypically abnormal B or T cell populations. Concurrent with these biopsies, the patient had thoracentesis and paracentesis for symptomatic relief. While lymphoma was suspected, the diagnosis was not confirmed, and HLH-specific treatment was instituted.

The patient was started on induction chemotherapy per the HLH-2004 protocol with etoposide and dexamethasone. However, after three days of treatment, she became febrile, tachycardic, and hypotensive and was started on broad-spectrum antibiotics. The inguinal node biopsy site was noted to be draining copious amounts of purulent material. Inflammatory markers, LFT, and ferritin continued to trend upward, and she was transferred to the intensive care unit for septic shock. Chemotherapy was held pending the resolution of sepsis. Unfortunately, despite appropriate intensive management, the patient continued to deteriorate and eventually expired.

## Discussion

As described in the case above, HLH can be extremely difficult to recognize, and even a slight delay in its diagnosis can lead to significant morbidity and mortality. Existing literature describes the rarity and severity of this condition but does not highlight how difficult it can be to diagnose and manage in practice. Most patients with HLH present with systemic inflammatory response syndrome (SIRS), which may point clinicians to believe the patient's presentation is an infectious process. This sepsis-like syndrome, when untreated in due time, may progress to terminal multiple-organ failure. In our case, the patient's disease process was advanced to the point where she had significant organomegaly and laboratory derangements, which guided clinicians toward a malignant process, but this may not always be the case.

Diagnostic criteria for HLH were first put forward in 1991 and referred to as the HLH-94 criteria [3]. These criteria divided the diagnosis of HLH into three separate categories: clinical criteria, laboratory criteria, and histopathological criteria. These criteria were revised in 2004 into the HLH-2004 criteria used today [4]. The HLH-2004 criteria introduced four further diagnostic criteria: hyperferritinemia, high interleukin-2-receptor levels (sCD25), low to absent natural killer (NK) cell activity, and elevated levels of the chemokine CXCL9. The revision further states that at least five out of nine (fever, splenomegaly, cytopenia, hypertriglyceridemia or hypofibrinogenemia, hemophagocytosis, hyperferritinemia, low to absent NK cell activity, elevated sCD25 and elevated CXCL9) criteria must be present. Moreover, if familial HLH is the cause, a molecular diagnosis solely is enough to diagnose with certainty. This involves mutation of an HLH-associated gene, such as PRF1/Perforin, UNC13D/Munc13-4, STX11/Syntaxin 11, STXBP2/Munc18-2, RHOG/RhoG, CDC42/Cdc42.

Based on the HLH-2004 criteria, this patient met six of nine criteria: fever, splenomegaly, cytopenias, hypertriglyceridemia, hyperferritinemia, and hemophagocytosis in the bone marrow. NK-cell activity, sCD25, and CXCL9 were unavailable for measurement in our institution. One prospective study demonstrated a higher proportion of activated cytotoxic CD4+ cells in HLH despite no triggering infection, possibly explaining its poor prognosis [5]. As seen in this case, severe immune system activation leads to rapid detrimental effects, with little time to establish a clear trigger for HLH. HLH disease is usually idiopathic, induced by an infection or a response to other immune system activation- such as an autoimmune disease or malignancy. In our patient, we suspected malignancy as the primary driver for her illness. Her inguinal node with CD20+ and CD 5+ lymphocyte proliferation suggested B-cell lymphoma. B-cell lymphoma accounts for 20-25% of cancer diagnoses in adolescents and young adults [6]. Lymphoma-associated hemophagocytic syndrome (LAHS) is a phenomenon that has previously been described. It is believed that LAHS has a worse prognosis than other forms of HLH, not solely due to the delay of lymphoma diagnosis and treatment [7]. However, a comparative study by Yu et al. shows that B-cell lymphomas had a better prognosis than T-cell lymphoma [8].

As mentioned above, differentiating sepsis from HLH can be highly challenging. However, as shown in the case, some laboratory criteria may help guide clinical decision-making. For example, progressive ferritinemia with levels > 2000 ng/ml in patients without a history of transfusion or defect of iron metabolism may suggest HLH. A hemoglobin concentration < 8 g/dL is more likely to be seen in HLH rather than sepsis, and thrombocytopenia is not usually seen in sepsis unless there is concomitant disseminated intravascular coagulation. Additionally, hypertriglyceridemia is a marker in HLH with relatively high specificity. Despite being separate entities, sepsis and HLH can overlap in the same presentation, known as sepsis-HLH overlap syndrome (SHLHOS). Our patient's source of infection seemed to have originated from her inguinal node biopsy site before spreading systemically. Our patient did not present in sepsis but developed septicemia after the initial presentation of HLH. Counterintuitively, immune system hyperactivation (as seen in HLH) makes patients more susceptible to worse outcomes with infection from a misdirection of immune system components. The cytokine storm (overproduction of interferon-gamma, chemokine CXCL9, TNF-alpha, and various interleukins) is thought to be responsible for multiorgan failure and leads to an inability to recover from superinfection [9]. Treating a patient whose HLH is triggered by infection or who is on the spectrum of SHLHOS may present a problem for clinicians. The HLH-2004 treatment protocol involves the use of steroids during induction, which has the potential to cause worsening of underlying sepsis. This is in addition to underlying pancytopenia and immune system defects. Initiation of HLH-specific therapy for severely ill patients should not be delayed while awaiting the resolution of systemic infection [10].

However, this should be assessed on a case-by-case basis. In our patients, despite extensive microbial coverage, sepsis is overwhelmed. This case underscores the difficulty in managing these very ill patients. The multidisciplinary decision was made to withhold immune-suppressing chemotherapy pending sepsis resolution.

## Conclusions

In patients with HLH, evaluation for underlying causes is as paramount as early detection of HLH itself. Early recognition and intervention, often involving immunosuppressive therapy and, in some cases, hematopoietic stem cell transplantation, is crucial for improving outcomes in individuals with HLH. Prompt initiation of HLH treatment takes precedence over treating its underlying cause; however, this is usually assessed individually and may vary based on the patient's condition.
